# Computational and Theoretical Analysis of Human Diseases Associated with Infectious Pathogens

**DOI:** 10.1155/2015/431706

**Published:** 2015-09-08

**Authors:** Suares Clovis Oukouomi Noutchie, Cedrick Aurelien Kitio Kwuimy, Jean Jules Tewa, Farai Nyabadza, Necdet Bildik

**Affiliations:** ^1^MaSIM Focus Area, North-West University, Mafikeng Campus, Mmabatho 2735, South Africa; ^2^Center for Nonlinear Dynamics and Control, Villanova University, Villanova, PA 19805, USA; ^3^National Advanced School of Engineering, University of Yaoundé I, BP 337, Yaoundé, Cameroon; ^4^Department of Mathematical Sciences, University of Stellenbosch, Stellenbosch 7600, South Africa; ^5^Department of Mathematics, Celal Bayar University, Muradiye Campus, 45047 Manisa, Turkey

Mathematical models and computer simulations are useful experimental tools for building and testing theories, assessing quantitative conjectures, answering specific questions, determining sensitivities to changes in parameter values, and estimating key parameters from data. Understanding the transmission characteristics of infectious diseases in communities, regions, and countries can lead to better approaches to decreasing the transmission of these diseases.

The present special issue aims at providing a platform for the discussion of major research challenges and recent achievements regarding the computational aspect of theoretical models describing human diseases associated with infectious pathogens. Appropriate mathematical models with new insights, relevant analysis, and validation with the help of numerical simulations are discussed.

Y. E. García et al. in Mexico in “A Bayesian Outbreak Detection Method for Influenza-Like Illness” considered a theoretical eruption detection method for influenza-like illness from surveillance data. It is well known that epidemic outbreak detection is an important problem in public health. The development of reliable methods for outbreak detection remains an active research area. During the early phase of the outbreak, surveillance data changes from autoregressive dynamics to a regime of exponential growth. Bayesian model selection and Bayesian regression are used to identify the breakpoint. In particular no free parameters need to be tuned and historical information regarding the spread of the disease is incorporated into the model. Furthermore synthetic, seasonal, and pandemic outbreak data are analysed in order to show and discuss the performance of the method.

F. Nyabadza et al. in South Africa and USA in “The Implications of HIV Treatment on the HIV-Malaria Coinfection Dynamics: A Modeling Perspective” investigated the scenario of hosts harbouring multiple pathogens at the same time in disease epidemiology. Multiple pathogens have the potential for interaction resulting in negative impacts on host fitness or alterations in pathogen transmission dynamics. They developed a mathematical model describing the dynamics of HIV-malaria coinfection. Additionally, they examined the role treatment (of malaria and HIV) plays in altering populations' evolution. The model consists of 13 interlinked equations describing multiple aspects of HIV-malaria transmission and treatment. Qualitative analysis of the model including positivity and boundedness of solutions was performed. Furthermore, reproduction numbers corresponding to submodels were evaluated and the long-term behaviour of the submodels was investigated.

Z. Shi et al. in USA in “Mathematical Model of Innate and Adaptive Immunity of Sepsis: A Modeling and Simulation Study of Infectious Disease” examined a mathematical model of a systemic inflammatory response to infection known as sepsis. Both innate and adaptive immunities are included, and simulated results have shown that adaptive immunity has significant impacts on the outcomes of sepsis progression. Further investigation has found that the intervention timing, intensity of anti-inflammatory cytokines, and initial pathogen load are highly predictive of outcomes of a sepsis episode. Sensitivity and stability analyses were carried out using bifurcation analysis to explore system stability with various initial and boundary conditions. The stability analysis suggested that the system could diverge at an unstable equilibrium; after perturbations the maximum release rate of tumour necrosis factor by neutrophil falls below a certain level. This finding conforms to clinical findings and the existing literature regarding the lack of efficacy of anti-TNF antibody therapy.

Other papers in this special issue cover applications of fractional differential equations arising in the modelling of human diseases associated with pathogens. In the papers “On the Mathematical Analysis of Ebola Hemorrhagic Fever: Deathly Infection Disease in West African Countries” and “Model of Break-Bone Fever via Beta-Derivatives,” A. Atangana et al. made use of a new derivative called beta-derivatives. They used novel fractional analytic methods to show the existence and uniqueness of the associated evolution equations. Stability of the steady states was investigated using Lyapunov methods. In particular, iteration methods were considered in order to approximate the solution and project its long-term behaviour. In the papers “Computational Analysis of the Model Describing HIV Infection of CD4^+^T Cells” and “Modelling the Aggregation Process of Cellular Slime Mold by the Chemical Attraction,” the authors made use of homotopy decomposition method (HDM) to find approximate solutions of nonlinear differential systems. Numerical solutions are given and some properties show evidence of biologically practical reliance on parameter values. The reliability of HDM and the reduction in computations give HDM a wider applicability.

The editorial board trust that the set of papers accepted in this special issue will provide a benchmark for future theoretical analysis in this fast evolving research area.

